# Molecular Precursor Route to Bournonite (CuPbSbS_3_) Thin Films and Powders

**DOI:** 10.1021/acs.inorgchem.1c02001

**Published:** 2021-08-12

**Authors:** Yasser T. Alharbi, Firoz Alam, Khaled Parvez, Mohamed Missous, David J. Lewis

**Affiliations:** † Department of Chemistry, The University of Manchester, Oxford Road, Manchester M13 9PL, U.K.; ‡ Department of Materials, The University of Manchester, Oxford Road, Manchester M13 9PL, U.K.; § School of Electrical and Electronic Engineering, The University of Manchester, Sackville Street, Manchester M13 9PL, U.K.

## Abstract

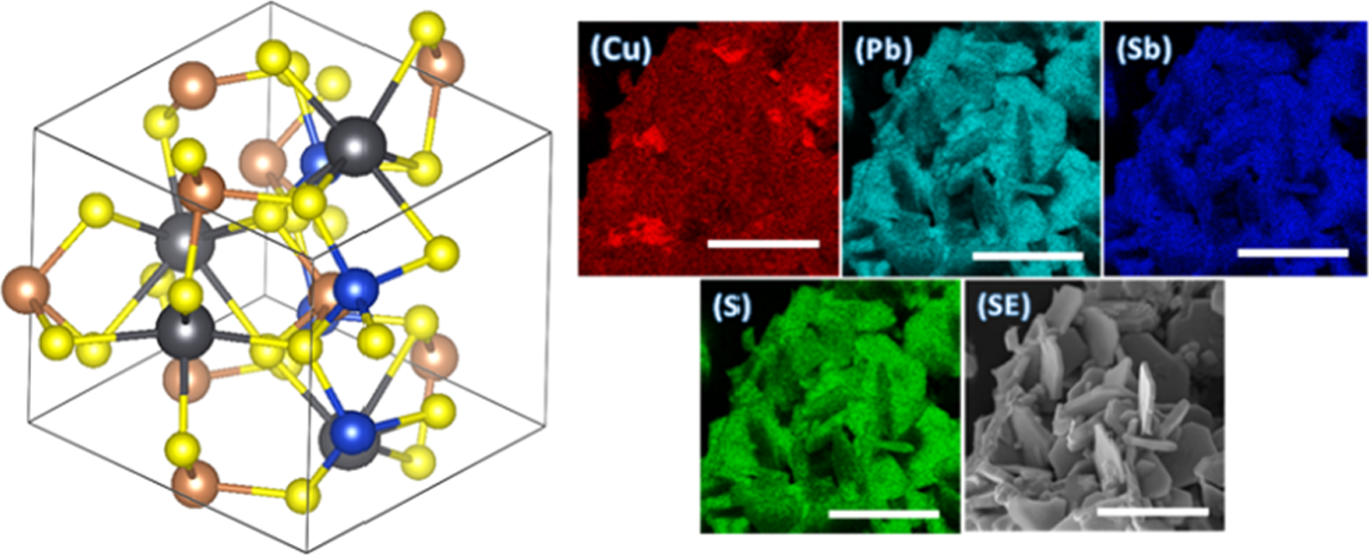

Quaternary metal chalcogenides have attracted attention as candidates for absorber materials for inexpensive and sustainable solar energy generation. One of these materials, bournonite (orthorhombic CuPbSbS_3_), has attracted much interest of late for its properties commensurate with photovoltaic energy conversion. This paper outlines the synthesis of bournonite for the first time by a discrete molecular precursor strategy. The metal dithiocarbamate complexes bis(diethyldithiocarbamato)copper (II) (Cu(S_2_CNEt_2_)_2_, (**1**)), bis(diethyldithiocarbamato)lead (II) (Pb(S_2_CNEt_2_)_2_, (**2**)), and bis(diethyldithiocarbamato)antimony (III) (Sb(S_2_CNEt_2_)_3_, (**3**)) were prepared, characterized, and employed as molecular precursors for the synthesis of bournonite powders and the thin film by solvent-less pyrolysis and spray-coat-pyrolysis techniques, respectively. The polycrystalline powders and thin films were characterized by powder X-ray diffraction (p-XRD), which could be indexed to orthorhombic CuPbSbS_3_. The morphology of the powder at the microscale was studied using scanning electron microscopy (SEM). Energy-dispersive X-ray spectroscopy (EDX) was used to elucidate an approximately 1:1:1:3 Cu/Pb/Sb/S elemental ratio. An optical band gap energy of 1.55 eV was estimated from a Tauc plot, which is close to the theoretical value of 1.41 eV.

## Abstract 2

Alharbi et al. showcase an inorganic molecular precursor approach to bournonite (copper lead antimony sulfide), which has attracted attention recently as a solar absorber material for energy generation via the photovoltaic effect.

## Introduction

Energy from fossil fuel is problematic as the source is inherently unsustainable and the products of combustion are associated with irreversible and dire climate outcomes.^1^ The search for alternative sustainable renewable energy sources is therefore one of the most important challenges faced by mankind currently.^2^ The development of solar power sources from energy conversion via the photovoltaic effect is an attractive sustainable solution to this problem. Predominantly mature technologies in this area are based on silicon, CIGS,^3^ or CdTe^4^ absorber materials. However, as the supply of elements is often governed by geopolitical factors,^5,6^ expanding the palette of efficient solar energy-generating materials is crucial to the sustainability of solar energy generation.^7^ In addition, the discovery of new processing routes to solar absorber materials is critical to allow compatibility with emerging non-traditional substrates, e.g., in flexible solar cells,^8^ and for producing tandem technologies.^9^

Metal chalcogenides are materials which can be potentially utilized for sustainable and inexpensive solar energy generation.^10^ These compounds can be categorized into three classes; (i) binary (M*_x_*E*_n_*), (ii) ternary (M*_x_*M^I^*_y_*E*_n_*), and (iii) quaternary (M*_x_*M*_y_*^I^M^II^E) metal chalcogenides, where M/M^I^/M^II^ are metals and E is a chalcogenide ion, i.e., S, Se, or Te.^11^ Quaternary chalcogenides based on naturally occurring minerals such as, for example, kesterite (tetragonal copper tin zinc sulfide, Cu_2_ZnSnS_4_, “CZTS”) have recently emerged as a class of promising compounds for use in solar energy generation applications due to their physical properties, which include band gaps commensurate with the capture of large amounts of solar flux combined with direct transitions that offer very high extinction coefficients.^12,13^ CZTS is also attractive because it is comprised of elements that are easy to obtain for most nations as mineral deposits and are inexpensive to process, allowing the prospect of energy security for many third world states. To reflect the larger research efforts in this area, Walsh and co-workers have recently begun to publish efficiency tables for inorganic solar cells,^7^ akin to the tables published by Green et al. that mainly consider the more mature single-junction technologies alongside established multijunction architectures.^14^

**Figure 1:**
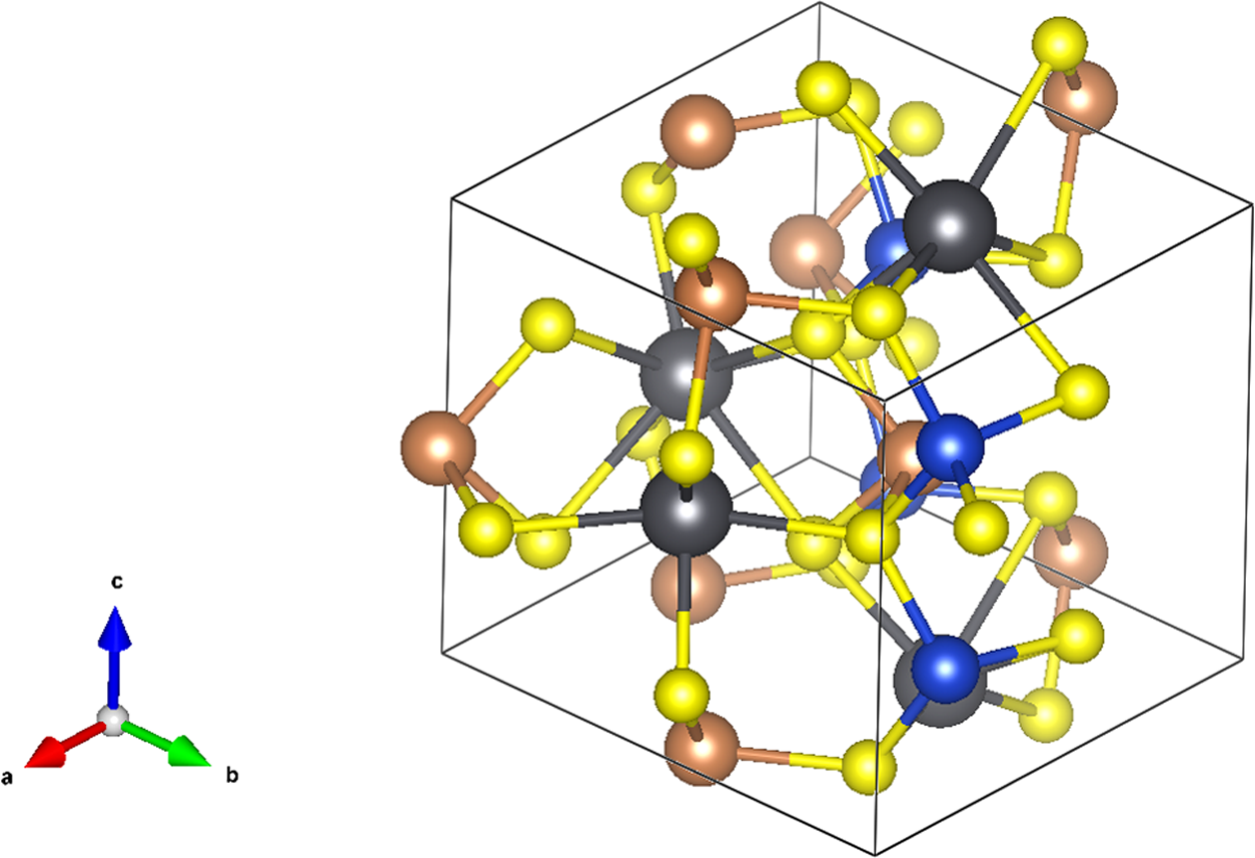
Structure of CuPbSbS_3_ (bournonite) with orthorhombic unit cell marked (*a* = 8.153 Å, *b* = 8.692 Å, and *c* = 7.793 Å with α = β = γ = 90°, *Pmn*2_1_) as reported by Edenharter et al.^16^ The blue atoms represent Cu, black atoms Pb, brown atoms Sb, and yellow atoms S.

The search for new materials for use in solar energy generation is ongoing to expand the portfolio of materials available for future terawatt energy generation from solar energy sources. Walsh et al. identified bournonite (copper lead antimony sulfide, CuPbSbS_3_), alongside enargite and stephanite, as the potential candidate photoferroic absorber material from 193 other candidates from search criteria that included natural occurrence, band gaps in the visible–near-infrared (vis–NIR) region of the electromagnetic spectrum, and potential ferroelectric characteristics via a polar crystal structure.^15^ The orthorhombic and non-centrosymmetric crystal structure of bournonite (Figure 1) can be envisaged approximately as the orthorhombic antimony sulfide structure with Pb(II) ions occupying alternating metal sites and the formation of charge-compensating Cu–S tetrahedra to form the neutral solid.^16^ Density functional theory (DFT) calculations of the bournonite structure using the FHI-aims all-electron structure code, which more accurately estimates optical properties, showed that the predicted band gap energy of this material was potentially commensurate with the capture of solar flux (*E*_g_ ∼ 1.37 eV) and was near to direct in nature, but in addition, the lowest energy direct transition occurred at 1.41 eV.^15^ The same group has recently reported candidate hole-transport and electron-transport materials paired specifically to bournonite.^17^ Bournonite has also been reported by Dong et al. to have a remarkably low thermal conductivity due to the stereochemically active s^2^ electrons on Pb and Sb; this property is fortuitous for thermoelectric energy generation.^18^ More recently, a study by Liu and co-workers has realized solar cells with bournonite as the absorber layer, which is formed from the constituent metal oxides dissolved in ethanolic carbon disulfide, with butylamine added to potentially form metal dithiocarbamate precursors in situ.^19,20^ However, there is a clear weakness in this route in that the precursors are not isolated and are thus ill-defined, which has potential ramifications in the resultant metal sulfide stoichiometry, which can be critical to device performance. Devices based on a p–i–n architecture of glass/ITO/CdS/CuPbSbS_3_/Spiro-OMeTAD/Au exhibited power conversion efficiencies of 2.23%, with high open-circuit voltages of 0.7 V, suggesting the material was relatively free of the defect trap states in the band gap that are commonly deleterious to metal chalcogenide performance in optoelectronic devices,^21,22^ although it is worth noting that surface engineering and doping strategies have been proposed to combat these effects.^23^ Indeed, by enhancement of the crystallinity of bournonite, Liu and co-workers have shown that it is possible to improve the power conversion efficiency of devices now to around 2.65% by reducing the carbon residue within the absorber layer via solvent tuning and by increasing the temperature of the annealing step to produce the films (380 °C was optimal in the context of device performance).^20^ Interestingly, in the latter study, the bournonite materials were found to be fairly stable after 1 month, aged in air with 86% of the original efficiency retained, and losses mainly from reduction in *J*_sc_.

The key then therefore to producing efficient solar devices is to produce pure phase bournonite that lacks significant contamination and has good crystallinity. However, the synthesis of bournonite is particularly refractory by solid-state methods due to the thermodynamic stabilities of both PbS and Sb_2_S_3_, which are often observed as crystalline byproducts. A chemical approach to the synthesis of bournonite nanocrystals has been reported by Nolas and co-workers from a mixture of lead (IV) acetate, copper (II) acetate, antimony (III) chloride, and elemental sulfur in olelylamine dissolved at 110 °C and then held at 280 °C for various time periods (5, 20, 60 min). Bournonite nanoparticles with sizes in the range of 3–240 nm could be produced by control of the heating step time. The synthesis of bournonite from amine–thiol alkahests containing dissolved solid-state metal oxides and metal chalcogenides has been pioneered by Brutchey and co-workers.^24^ Thin films were produced from solutions of bulk CuO, PbO, and Sb_2_S_3_ precursors in a mixture of ethylene diamine and 1,2-ethanedithiol. The alkahest solutions were spin-coated onto substrates, followed by a thermal annealing step at 450 °C. An optical band gap of 1.24 eV and a high absorption coefficient in the visible range of ∼10^5^ cm^–1^ were reported. It was noted that extended annealing times (> 60 min) resulted in the formation of Sb_2_S_3_ and PbS via disproportionation. Interestingly, the bournonite could also be delivered via this solution pathway by direct dissolution of a natural mineral sample of bournonite dissolved directly in the thiol–amine alkahest, followed by drop-casting of the solution and thermal annealing; however, the latter samples had significant impurities from PbS as inclusions in the deposited material.

The use of precursor chemistry, where a set of discrete molecular precursors are used to produce bournonite has, to the best of our knowledge, not been reported thus far. However, such a route would have a number of advantages for the synthesis of bournonite absorber layers. First, the metal stoichiometry can be easily controlled by judicious mixing of precursors in the required ratios. We have recently, for example, reported the synthesis of ternary copper iron sulfide thin films by simple thermolysis of a mixture of Cu and Fe xanthates.^25^ We have also demonstrated that mixing of metal dithiocarbamate precursors allows the spray-coating of metal sulfide thin films including examples of binary (1 precursor), ternary (2 precursors), and quaternary (3 precursors) metal sulfide materials (*n* = 18).^26^ For the synthesis of bournonite, this mixed precursor approach could be used to easily explore off-stoichiometric materials and doped and alloyed derivatives, which are known or predicted to exist.^24,27^ Second, molecular precursors tend to form kinetic products due to the rapid decomposition and ultimate crystallization steps involved, which could sidestep the potential formation of PbS and Sb_2_S_3_ side products.^18,24^ We have recently observed the formation of kinetic products, for example, in the formation of molybdenum–tungsten trioxide alloys.^28^ In this paper, we explore for the first time the use of metal dithiocarbamate complexes as precursors toward bournonite. We show that the production of powders is possible via direct thermolysis of precursor mixtures, and then we show that this principle can be adapted for spray-coating of precursors onto substrates that after thermal annealing produce bournonite films.

## Experimental Section

### Chemicals

Methanol (CH_3_OH, 99.8%), chloroform (CHCl_3_, ≥99%), copper (II) chloride (CuCl_2_, ≥99.9%), antimony (III) chloride (SbCl_3_, ≥99.9%), lead (II) acetate trihydrate (Pb(CH_3_CO_2_)_2_·3H_2_O, 99.9%), and sodium diethyldithiocarbamate trihydrate ((C_2_H_5_)_2_NCSSNa·3H_2_O) were purchased from Sigma-Aldrich and used without further purification.

### Instrumentation

Melting points were measured using a Barloworld SMP10 apparatus. Infrared (IR) spectra were recorded using a Specac single reflectance ATR instrument (in the range 4000–400 cm^–1^, resolution 4 cm^–1^). Elemental analysis of the precursors was obtained by the chemistry microanalysis laboratory at the University of Manchester. ^1^H and ^13^C NMR spectra were obtained using a Bruker AC400 FT-NMR spectrometer. Thermogravimetric analysis (TGA) measurements of the precursors were performed using a Seiko SSC/S200 model under a heating rate of 10 °C min^–1^ under nitrogen. X-ray diffraction (XRD) measurements were performed using a Bruker D8 Advance diffractometer equipped with a Cu Kα radiation source (λ =1.5406 Å) with a step size of 0.02°. Raman spectra were obtained using a Renishaw 1000 microscope system equipped with laser excitation of 514 nm. Absorption spectra were recorded using a Shimadzu UV-1800 instrument. Scanning electron microscopy (SEM) images and energy-dispersive X-ray spectroscopy (EDX) were obtained using a Tescan SC with an Oxford Instruments EDX detector.

### Synthesis

#### Bis(diethylthiocarbamato)copper (II), Cu(S_2_CN(C_2_H_10_)_2_)_2_ (**1**)

An aqueous solution of CuCl_2_ (1.0 g, 7.43 mmol, 1 equiv) was added dropwise to an aqueous solution of Na(S_2_CNEt_2_) (3.34 g, 14.86 mmol, 2 equiv) with stirring. The mixture was left to stir for 1 h at room temperature. The product obtained was filtered and washed with deionised water. After that the product was recrystallized from 1:1 v/v of dichloromethane (DCM) and EtOH to obtain the title product. Finally, black crystals were formed after drying at room temperature overnight. Yield, 2.0 g (83%); melting point (mp), 210–212 °C. Element analysis: calcd (%): C, 33.37; H, 5.61; N, 7.79; S, 35.11; Cu, 17.67; found (%): C, 33.40; H, 5.57; N, 7.7; S, 35.52; Cu, 17.5. Fourier transform infrared (FT-IR) (solid) (ν_max_/cm^–1^): 2973 (m), 1460 (s), 1434 (s), 1277 (s), 1202 (s), 1145 (s), 990 (s), 838 (m), 773 (m), and 565 (m).

#### Bis(diethylthiocarbamato)lead (II), Pb(S_2_CN(C_2_H_10_)_2_)_2_ (**2**)

Lead acetate (2.0 g, 6.14 mmol) was dissolved in 20 mL of deionised water and then added dropwise to a 25 mL aqueous solution of Na(S_2_CNEt_2_) (2.1 g, 12.2 mmol) under stirring. The reaction was left under constant stirring at room temperature for 35 min. A white precipitate was formed and isolated by filtration at room temperature. Yield 2.8 g (87.5%), mp 210 °C. Elemental analysis: found (%): C 24.14; H 4.05; S 25.53; N 5.48; Pb 41.60; calcd (%) C 23.84; H 4.01; S 25.41; N 5.47; Pb 41.17. FT-IR (solid) (ν_max_/cm^–1^): 2964 (m), 2929 (m), 1480 (s), 1457 (s), 1351.37 (s), 1298 (m), 1264 (s), 1200 (s), 1075 (s), 980(s), 565(s).

#### Tris(diethylthiocarbamato)antimony (III), Sb(S_2_CN(C_2_H_10_)_2_)_3_ (**3**)

Antimony trichloride (2.0 g, 8.766 mmol) was dissolved in 20 mL of ethanol under constant stirring at room temperature. Na(S_2_CNEt_2_) (4.5 g, 26.27 mmol) was also dissolved in 40 mL of ethanol and then added dropwise to the antimony trichloride solution in ethanol; stirring was continued for 1 h. A yellow precipitate was formed immediately and collected by vacuum filtration and dried at room temperature overnight. Yield 3.9 g (75.5%), mp 275 °C. Elemental analysis: found (%): C 31.98; H 5.37; S 33.86; N 7.35; Sb 41.19; calcd (%): C 31.81; H 5.34; S 33.90; N 7. 42; Sb 21.20. FT-IR (solid) (ν_max_/cm^–1^): 2985 (m), 2932 (w), 1488 (vs), 1431 (vs), 1374.51 (m), 1350 (s), 1295 (m), 1264 (vs), 1203 (s), 1071(s), 988 (s), 837.92 (s), 666.29 (m), 560 (vs).

### Synthesis of M(S_2_CN(C_2_H_10_)_2_)*_n_* (where M = Cu, Pb, and Sb)

The precursors were synthesized according to the literature.^29−31^ Briefly, dithiocarbamate complexes of Cu^2+^, Pb^2+^, and Sb^3+^, were synthesized by metathesis of the metal chloride or metal acetate with sodium diethyldithiocarbamate to furnish M(S_2_CN(C_2_H_10_)_2_)*_n_* (where M = Cu, Pb, and Sb).

### Synthesis of Bournonite Powders by Direct Thermolysis of Precursors

Copper (II) diethylthiocarbamate (0.10 g, 0.27 mmol), lead (II) diethylthiocarbamate (0.13 g, 0.25 mmol), and antimony (III) diethylthiocarbamate (1.5 g, 0.26 mmol) with a molar ratio of 1:1:1 were mixed homogeneously together and loaded into a ceramic boat. The loaded ceramic boat was subsequently placed in the center of a glass tube, which was then inserted into a carbolite tube furnace. The mixture was heated at 400 and 500 °C for 1 h under nitrogen atmosphere to produce CuPbSbS_3_ powders. The product was collected after cooling to room temperature.

### Synthesis of Bournonite Thin Films by Spray-Coat-Pyrolysis

A spray-coat-pyrolysis method was used to deposit thin films. Precursors (**1**), (**2**), and (**3**) in a molar ratio 1:1:1 were dissolved in tetrahydrofuran (THF, 20 mL) by stirring for 35 min. The magazine of an artistic airbrush was then loaded with the precursor solution, which was then air spray onto a preheated cleaned glass substrate at 180 °C. After drying for some time, a homogeneous and uniform film appeared on the glass substrate. After that the deposited films were loaded into a carbolite tube furnace and heated for 1 h at 400 and 500 °C under argon over the period of pyrolysis. After cooling down to room temperature, dark black films were collected for further analysis.

## Results and Discussion

**Figure 2:**
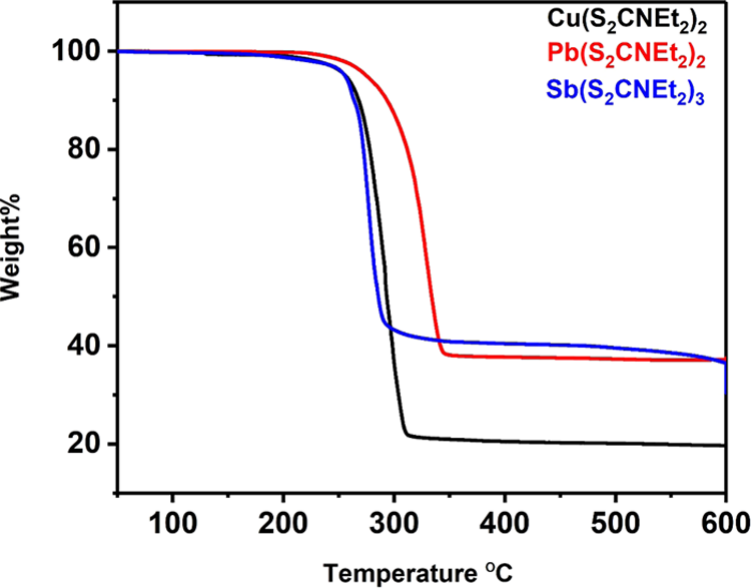
TGA profiles of Cu(S_2_CNEt_2_)_2_ (**1**), Pb(S_2_CNEt_2_)_2_ (**2**), and Sb(S_2_CNEt_2_)_3_ (**3**).

Thermogravimetric analysis (TGA) was used to investigate the thermal decomposition profiles of the precursors Cu(S_2_CNEt_2_)_2_ (**1**), Pb(S_2_CNEt_2_)_2_ (**2**), and Sb(S_2_CNEt_2_)_3_ (**3**) in the temperature range of 50–600 °C with a heating rate of 10 °C min^–1^ under a nitrogen atmosphere. The TGA profiles generated (Figure 2) show the decomposition of the metal diethyldithiocarbamate to form metal sulfides. Both complexes (**1**) and (**2**) show a one-step decomposition, while complex (**3**) shows a three-step decomposition. The total mass recorded for the Cu(S_2_CNEt_2_)_2_ precursor after heating at 331 °C was 21%, which corresponds to the calculated value for Cu_2_S (21%). In addition, the Pb(S_2_CNEt_2_)_2_ complex exhibited complete decomposition around 355 °C with a weight loss of 38% and a theoretical value of 47%. The small difference between the experimental and theoretical values is due to the poor volatility of residues in some transition-metal dithiocarbamate precursors.^32,33^ Finally, the decomposition of the Sb(S_2_CNEt_2_)_3_ complex occurred in two minor steps (10, 8%) and one major step (51%), with a total weight loss of 31%, which is close to the theoretical value of 30%, consistent with formation of Sb_2_S_3_. We also note that Sb_2_S_3_ is volatilized with the increase in temperature above 580 °C, probably due to loss of sulfur as the step is slow and steady as a function of temperature.

Powder X-ray diffraction (P-XRD) was used to study the crystalline components of the residues for each of the precursors heated at 400 and 450 °C for 1 h under nitrogen. The final products were black powders for M(S_2_CNEt_2_)_*n*_ (where M = Cu, Pb, and Sb). The p-XRD pattern of products synthesized from complexes (**1**), (**2**), and (**3**) confirms the formation of tetragonal Cu_1.96_S, cubic PbS, and orthorhombic Sb_2_S_3_, respectively. The results of the p-XRD, SEM, and EDX analyses for all of the binary metal sulfides obtained are shown in the Supporting Information.

### Synthesis and Characterization of Bournonite Powders

**Figure 3:**
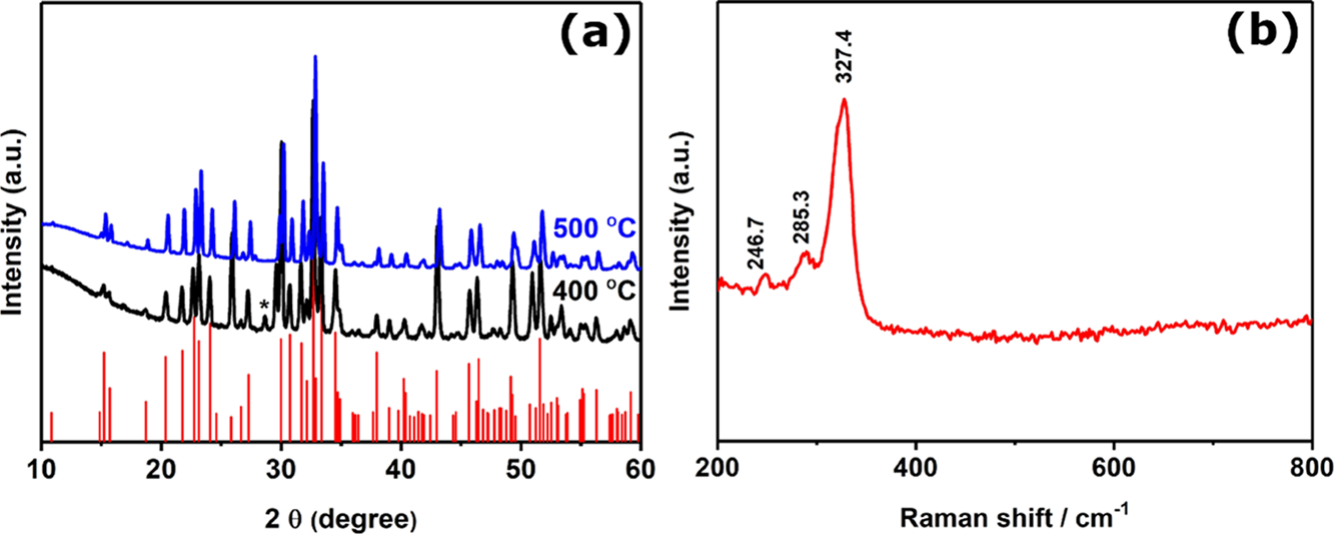
(a) p-XRD patterns of the bournonite (CuPbSbS_3_) powder synthesized at 400 and 500 °C for 1 h. The standard pattern confirmed orthorhombic bournonite, CuPbSbS_3_ (ICDD No. 01-076-1999), which is represented by red sticks. The black asterisk (*) refers to the minor reflections from Sb_2_S_3_ (ICDD No. 01-078-1347). (b) Raman spectrum of the sample synthesized at 500 °C showing Raman peaks at 246.7, 285.3, and 327.4 cm^–1^ corresponding to orthorhombic bournonite.

To produce bournonite powders, the three Cu, Pb, and Sb metal-containing dithiocarbamates were mixed together and heated in a Carbolite furnace at 400 or 500 °C for 1 h under nitrogen atmosphere. The black powder thus obtained was characterized using p-XRD and the diffraction pattern is shown in Figure 3a. The diffraction peaks observed at 2θ values correspond to the planes of orthorhombic bournonite (ICDD No. 01-076-1999) as shown in Supporting Information Table S1. The XRD peak intensity increases with the increase in temperature from 400 to 500 °C, which suggests that further growth of crystalline domains occurs at more elevated temperatures and greater crystallinity in the products. We note the growth temperature of 500 °C was optimal to overcome the minor reflections arising from cubic copper antimony sulfide (Cu_11_Sb_4_S_13_; ICDD No. 01-075-2219) at 16.5° and orthorhombic antimony sulfide (Sb_2_S_3_) (ICDD No. 01-078-1347) at 28.7° that were observed in the product from the synthesis at 400 °C (Figure 3a). Figure 3b shows the Raman spectrum of the bournonite phase synthesized at 500 °C, which has a dominant peak at 327 cm^–1^ and minor peaks centered at 285 and 246 cm^–1^. The high-intensity peak at 327 cm^–1^ has been assigned to vibrations from SbS_3_ groups in the bournonite structure.^34^ The relevant NMR data of the complexes used are shown in the SI.

**Figure 4:**
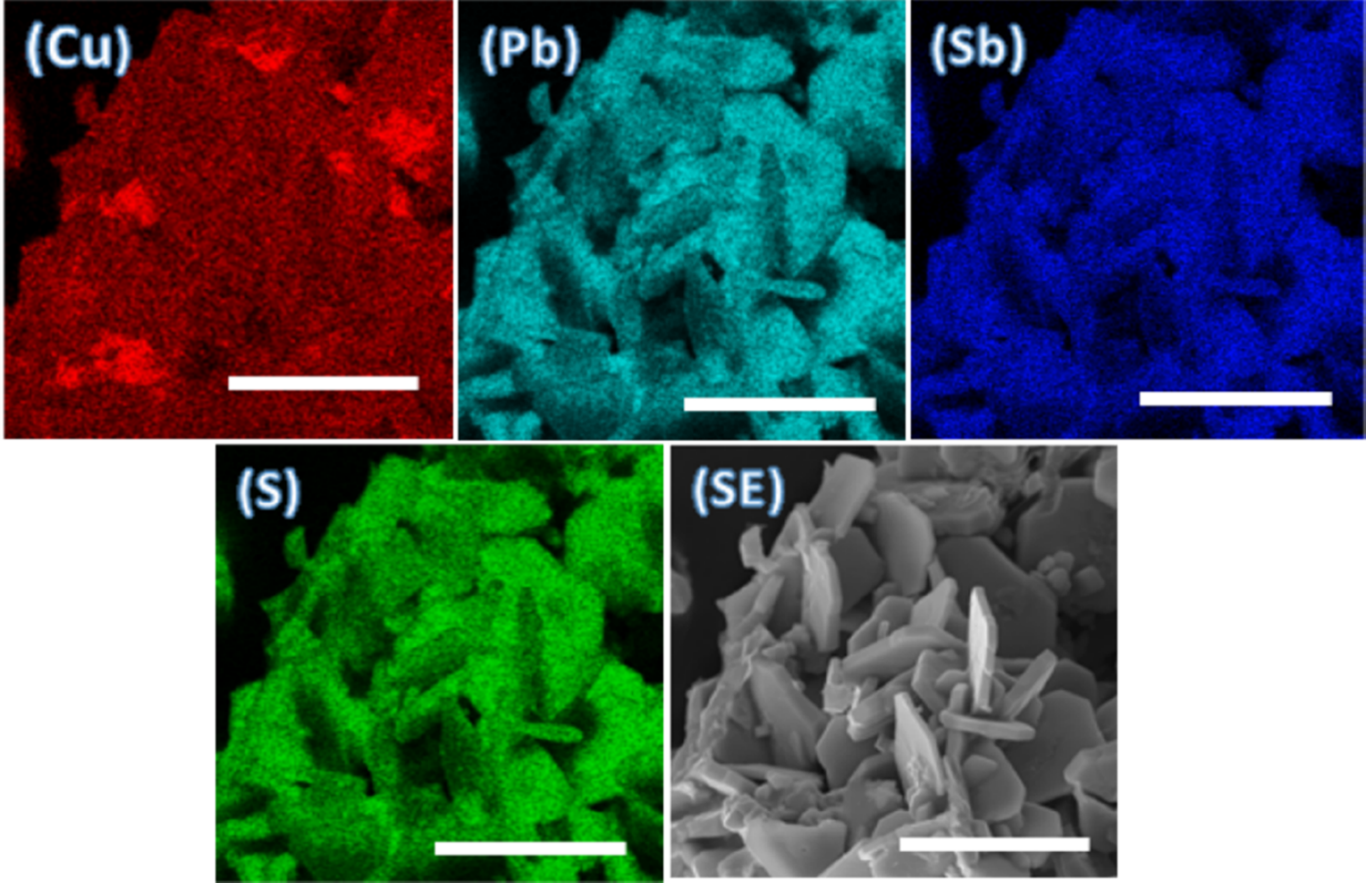
EDX elemental mapping of bournonite powders synthesized at 500 °C showing the spatial distribution of Cu, Pb, Sb, and S. Scale bars represent 10 μm in all cases.

Scanning electron microscopy (SEM) images and EDX mapping were performed to inspect the microscale features of the crystals as well as to elucidate the composition and spatial distribution of elements in the samples. The SEM images revealed agglomerates with sheet-like morphology as shown in Figure 4. The composition and stoichiometry of the as-synthesized bournonite was confirmed by EDX spectroscopy (see Supporting Information Figure S11b). The atomic % of Cu, Pb, Sb, and S were 18.5, 16.7, 14.8, and 50 atom %, respectively, which indicate that the bournonite phase of the CuPbSbS_3_ powder is near-stoichiometric. EDX mapping at the microscale is used to investigate the spatial distribution of elements (Figure 4). The Cu, Pb, Sb, and S elements in the sample were found to be fairly uniformly distributed at the microscale, which suggests that the single phase of bournonite is formed, which is consistent with both the X-ray diffraction and Raman spectroscopy data for this sample.

### Spray-Coat Pyrolysis of Bournonite Thin Films

**Figure 5:**
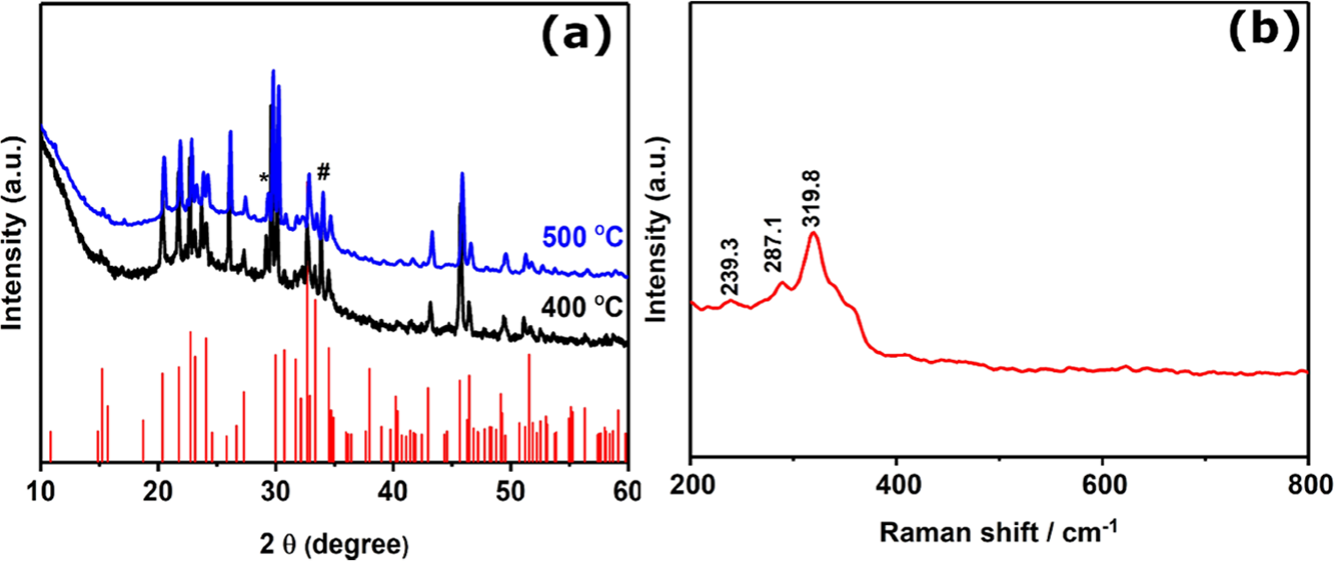
(a) p-XRD patterns of the bournonite film deposited on the glass substrate via spray-coat-pyrolysis and heated at 400 and 500 °C for 1 h. The pattern is matched to orthorhombic bournonite (ICDD No. 01-076-1999). The peak labeled with the asterisk (*) refers to cubic lead sulfide (PbS) (ICDD No. 03-065-2935) and the peak labeled with the hash (#) indicates the presence of the higher sulfide CuPb_13_Sb_7_S_24_ (00-043-1476). (b) Raman spectrum of bournonite deposited on the glass substrate by spray-coat-pyrolysis of precursors followed by heating at 500 °C.

As mentioned in the Introduction section, in this research we have recently turned our attention to using spray-coating to coat large-area substrates very rapidly with precursor molecules. When these molecules are subjected to thermal stress, they are found to decompose to metal sulfides.^26^ In this section of the paper, we now look at the application of this method, which has great potential for scalable deposition as well as the ability to coat complex substrates for deposition of bournonite films. The p-XRD pattern of films deposited by spray-coating followed by annealing at 400 and 500 °C confirms the orthorhombic bournonite phase (ICDD No. 01-076-1999). The diffraction peaks observed at 2θ values corresponding to planes of orthorhombic bournonite are shown in Supporting Information Table S2. We note that minor reflections of lead sulfide (PbS) (ICDD No. 03-065-2935) are observed at 29° as shown in Figure 5a. We also observe a reflection at 34°, which we ascribe to the orthorhombic meneghinite phase (CuPb_13_Sb_7_S_24_, ICDD No. 00-043-1476).

The Raman spectrum of the sample produced at 500 °C shows three prominent peaks (Figure 5b). The peak at 319.8 cm^–1^ was more intense than the peaks at 239.3 and 287.1cm^–1^, which agrees with the literature Raman spectra.^34^

**Figure 6:**
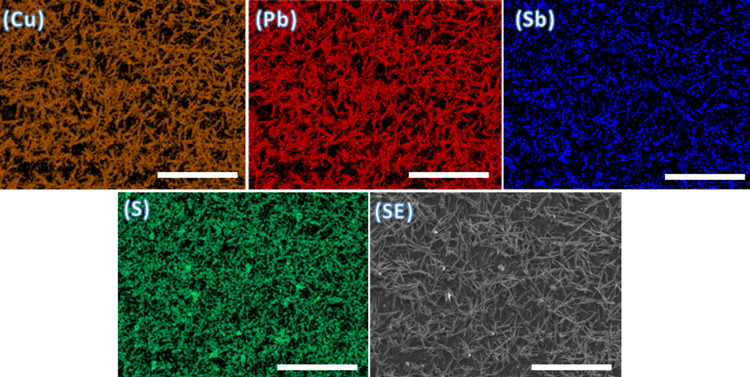
EDX spectrum maps of the Cu (Kα), Pb (Kα), Sb (Kα), and S (Kα) emission lines in a bournonite thin film deposited via spray-coating of precursors and heating at 500 °C for 1 h. The panel designated “SE” indicates the secondary electron image taken from the area being interrogated showing the morphology of the sample in plan view taken at the same length scale as the EDX spectrum maps. The scale bars represent 50 μm in all cases.

Figure 6 shows the SEM images of bournonite films deposited at 500 °C onto glass substrates by spray-coat-pyrolysis. We observe two different types of particles, namely, microsized octahedra embedded into wires, which are randomly distributed over the substrate (see Supporting Information Figure S12a for higher-magnification image of both particle types), whose diameters are in the nanometer range and length is in micrometers. The energy-dispersive X-ray (EDX) spectrum of the bournonite film is shown in Figure S12b, which shows the presence of Cu, Pb, Sb, and S in the film. The atomic % of Cu, Pb, Sb, and S were 13.8, 17.2, 14.5, and 54.5 atom %, respectively, which indicate that the bournonite phase of the CuPbSbS_3_ film is near-stoichiometric. The EDX mapping of the bournonite film demonstrated that copper, lead, antimony, and sulfide are uniformly distributed throughout the film at this length scale (Figure 6).

### Optical Properties of Bournonite Films

**Figure 7:**
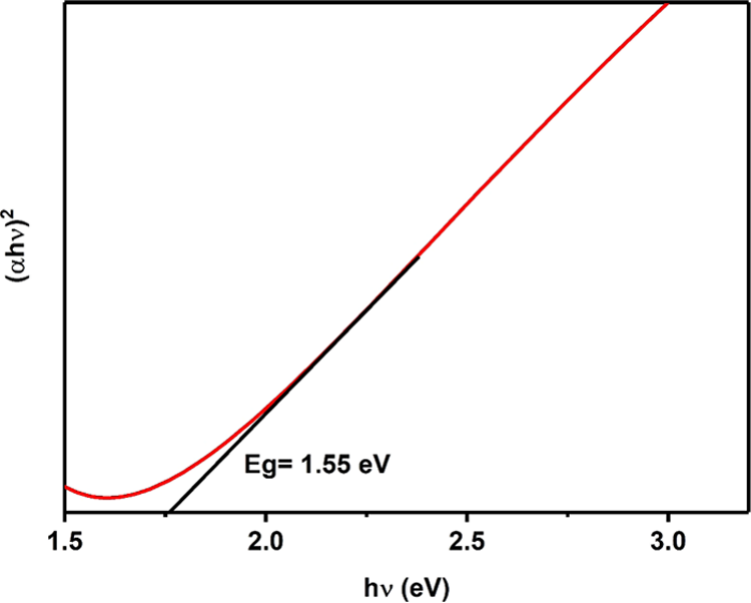
Tauc plot obtained from UV–vis absorption data of a bournonite film showing a direct band gap energy of 1.55 eV.

The absorption spectrum of the bournonite film was recorded in the wavelength range of 350–900 nm as shown in Figure S13. For this optical study, a bournonite film was prepared by dispersing the powders in the THF solvent and then the mixture was drop-casted onto a glass substrate. The direct optical band gap value of the CuPbSbS_3_ film was estimated using the Tauc method (α*h*υ)^2^ vs *h*υ as shown in Figure 7. The linear part of the Tauc plot was extrapolated to the energy (*h*υ) axis intercept to determine the direct optical band gap. The obtained value of 1.55 eV is close to the value of 1.41 eV reported by Walsh et al. for the lowest direct transition derived by DFT, and is closer than that reported by Brutchey (1.24 eV).^24^ Additionally, to know if the bournonite (CuPbSbS_3_) film is electrically active, we studied the electrical properties of the bournonite (CuPbSbS_3_) film using a four-point probe measurement. The details of conductivity measurements and electrical properties of bournonite (CuPbSbS_3_) films are shown in Table S3.

## Conclusions

Copper, lead, and antimony diethyldithiocarbamate molecular precursors have been synthesized, characterized, and used as single-source precursors for the synthesis of bournonite powders and thin films using the solvent-less and spray-coat-pyrolysis methods at 400 and 500 °C, respectively. The p-XRD patterns confirm the bournonite CuPbSbS_3_ phase from both the methods. The SEM images of the powders show that the crystallites have sheet-like morphology and there were no significant changes in morphology noticed when the growth temperature was increased. On the other hand, the thin films deposited onto glass substrates by the spray-coat-pyrolysis method show two shapes, octahedra and nanowires. Hence, both of the techniques produce different morphologies of the same material processed at the same temperature. EDX mapping of bournonite films deposited by the spray-coat-pyrolysis method onto the glass substrate demonstrated that copper, lead, antimony, and sulfur are uniformly distributed throughout the film. The Raman spectra of the materials also confirm the bournonite CuPbSbS_3_ phase. The band gap value of 1.55 eV indicates that the bournonite produced by this method can also potentially be used as an absorber material in thin film solar cells. The technique used to obtain CuPbSbS_3_ powders and thin films is simple, cost-effective, and has great potential for scale up with exquisite control over elemental stoichiometry in the final products.

## Supporting Information Available

^1^H and ^13^C NMR data; photographs of thin films; XRD patterns/Raman spectra/EDX analysis of binary chalcogenides; 2θ values for bournonite XRD peaks; elemental quantification of bournonite films by EDX; and UV–vis–NIR absorbance spectra and electrical transport data including sheet resistance and conductivity data from four-point probe measurement (PDF)
