# Supplementary material for: Molecular Precursor Route to Bournonite (CuPbSbS3) Thin Films and Powders
Source: Inorg Chem. 2021 Aug 12;60(17):13691–8. doi: 10.1021/acs.inorgchem.1c02001 (PMC8424643; doi:10.1021/acs.inorgchem.1c02001)
Supplement: Supplementary file 1 — ic1c02001_si_001.pdf [file ic1c02001_si_001.pdf]

Electronic supporting information (ESI)

A molecular precursor route to bournonite ($CuPbSbS_3$) thin films and powders.

Yasser T. Alharbi[a], Firoz Alam[a], Khaled Parvez[a], Mohamed Missous[c] and David J. Lewis[b]*

[a] *Department of Chemistry, The University of Manchester, Oxford Road, Manchester M13 9PL, UK.*

[b] *Department of Materials, The University of Manchester, Oxford Road, Manchester M13 9PL, UK.*

[c] *School of Electrical and Electronic Engineering, The University of Manchester, Sackville Street, Manchester, M13 9PL, UK.*

*Corresponding author: Dr: David Lewis; Email: david.lewis-4@manchester.ac.uk

^{1}H and ^{13}C NMR data for the lead and antimony complexes:

Bis(diethylthiocarbamato) lead(II), $Pb(S_2CN(C_2H10)_2)_2$

^{1}H NMR (400 MHz, $CDCl_3$ δ: 3.78 (q, J = 7.1 Hz, 2H), 1.32 (t, J = 7.1 Hz, 3H). ^{13}C NMR (101 MHz, CDCl3) δ: 202.09 (N$\underline{C}S_2$), 47.30 (N$\underline{C}H_2$ –), 12.28 (CH_3–).

Tris(diethylthiocarbamato) antimony(III), $Sb(S_2CN(C_2H_{10})_2)_3$

^{1}H NMR (400 MHz, $CDCl_3$ δ: 3.86 (q, J = 7.1 Hz, 2H), 1.29 (t, J = 7.1 Hz, 3H). ^{13}C NMR (101 MHz, CDCl3) δ 199.21(N$\underline{C}S_2$), 48.52(N$\underline{C}H_2$ –), 12.49(CH_3–).

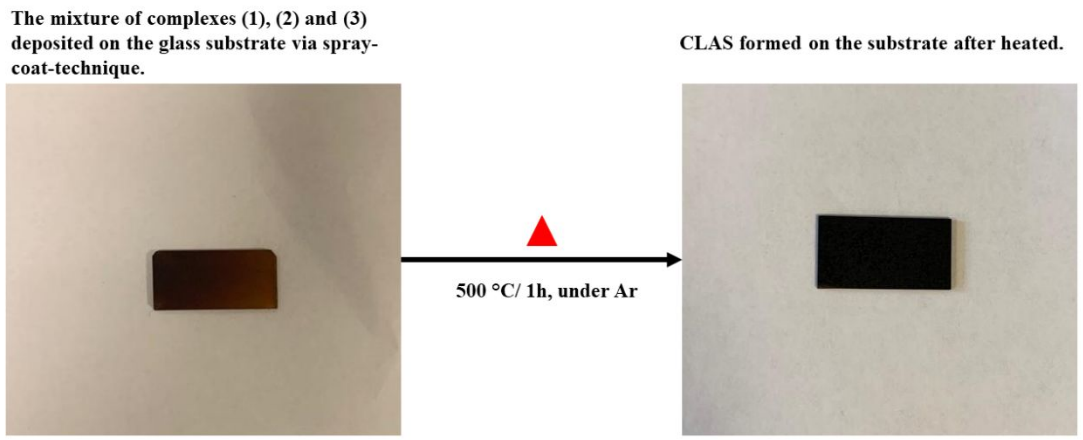

Figure S1: The left column shown the dissolved mixture of complexes (**1**), (**2**) and (**3**) deposited on the glass substrate via spray-coat-pyrolysis method. In the right shown the formation of quaternary material of bournonite after annealing at 500 °C under N_2.

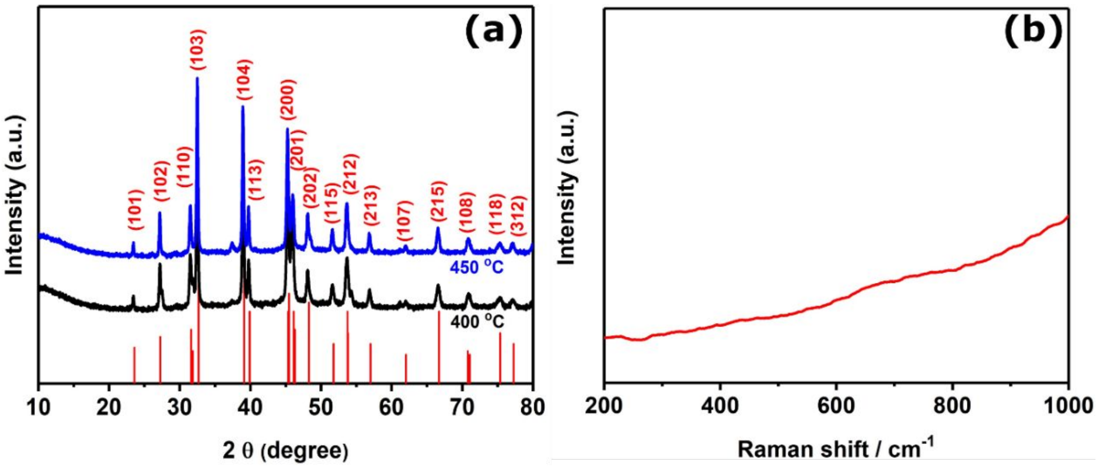

Figure S2 (a) shows p-XRD pattern of the copper sulfide nanocrystal produced from the decomposition of $Cu(S_2CNEt_2)_2$ complex (**1**) by solvent-less pyrolysis at 400 °C and 450 °C for 1 h. The XRD patterns confirmed the tetragonal copper sulfide (ICDD No. 00-029-0578), (b) Raman spectrum of copper sulfide nanocrystals produced at 450 °C for 1 h under N_2.

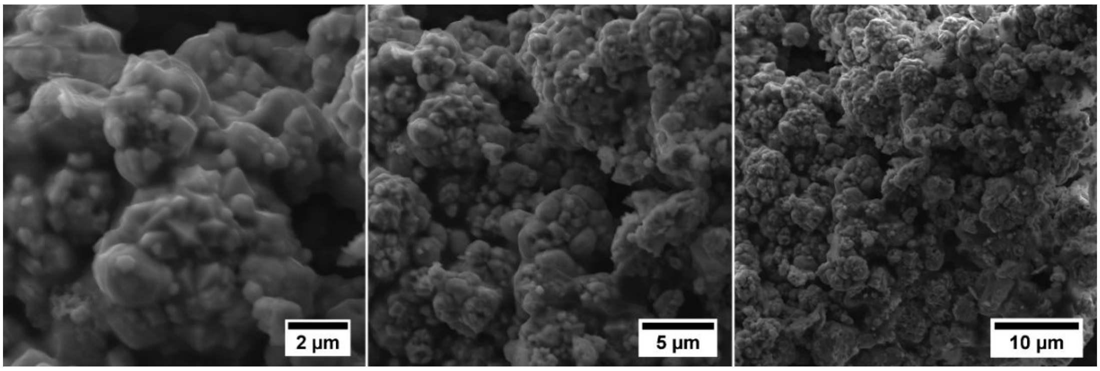

Figure S3: SEM images of copper sulfide nanocrystals synthesised at 450 °C for 1h under N_2 shows agglomerated spherical nanocrystals.

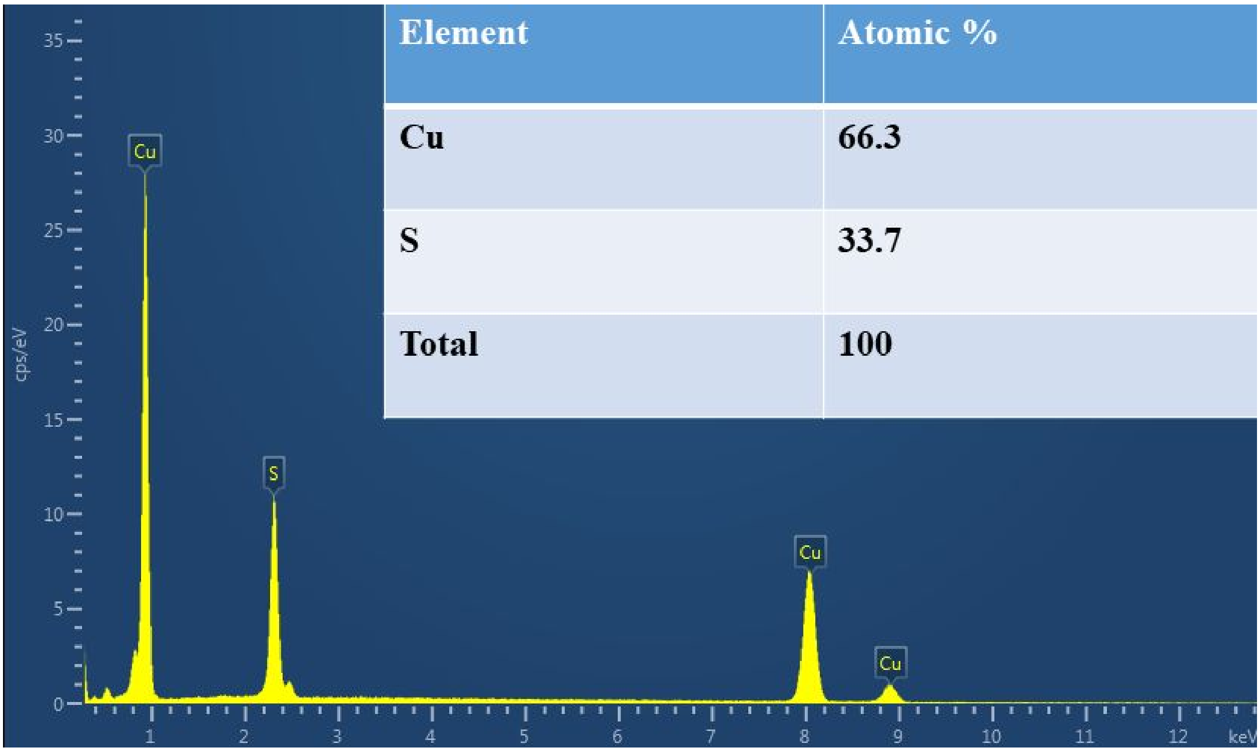

Figure S4: EDX plot of copper sulfide nanocrystals synthesised at 450 °C for1 h under N_2. Inset is the atomic percentage of Cu and S.

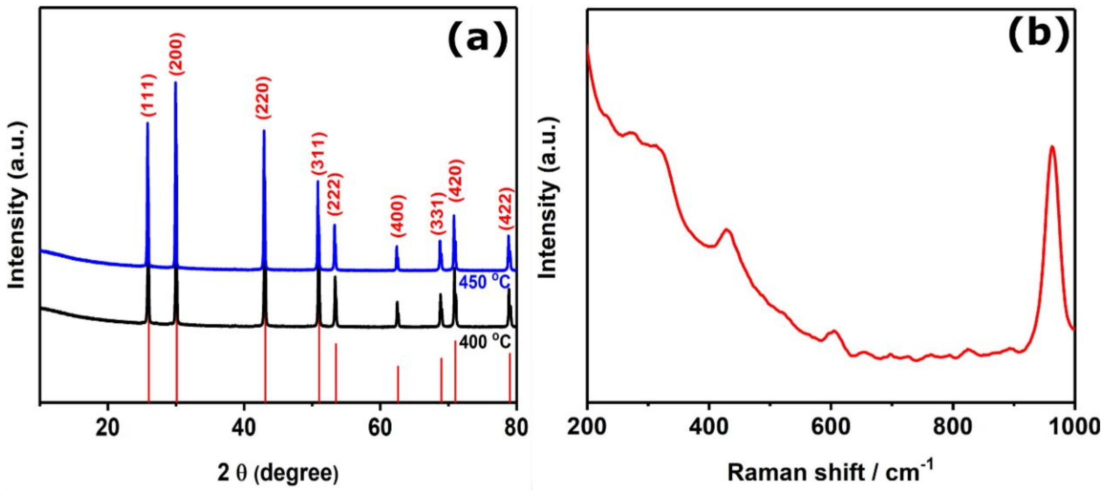

Fig S5 (a) shows p-XRD pattern of the PbS nanocrystal produced from decomposition of $Pb(S_2CNEt_2)_2$ complex (**2**) at 400 °C and 450 °C by solvent-less pyrolysis for 1 h under N_2. The XRD patterns confirmed the cubic lead sulfide (ICDD No. 03-065-0135) from $Pb(S_2CNEt_2)_2$ complex (**2**) and (b) Raman spectrum of PbS nanocrystals synthesised at 450 °C for 1 h under N_2.

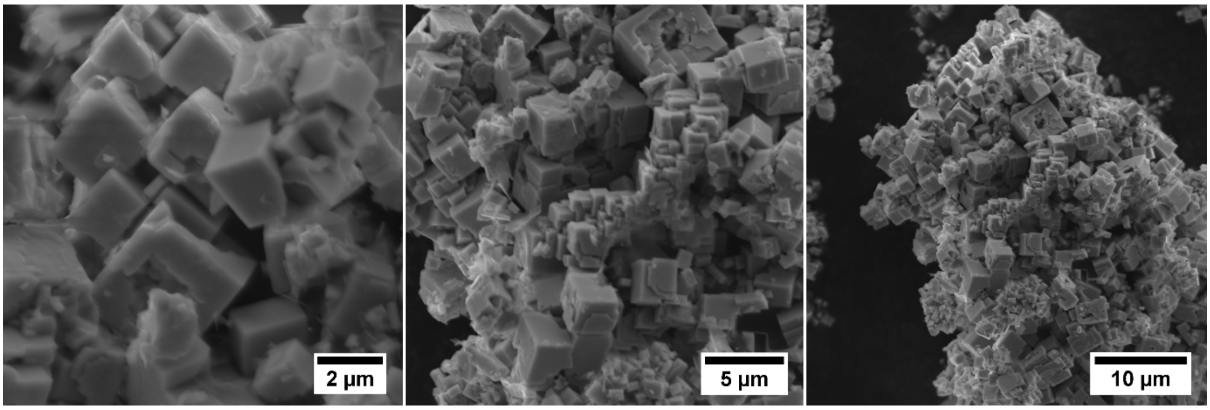

Fig S6 SEM images of lead sulfide nanocrystals synthesised at 450 °C for 1 h under N_2, which shows cubic nanocrystals.

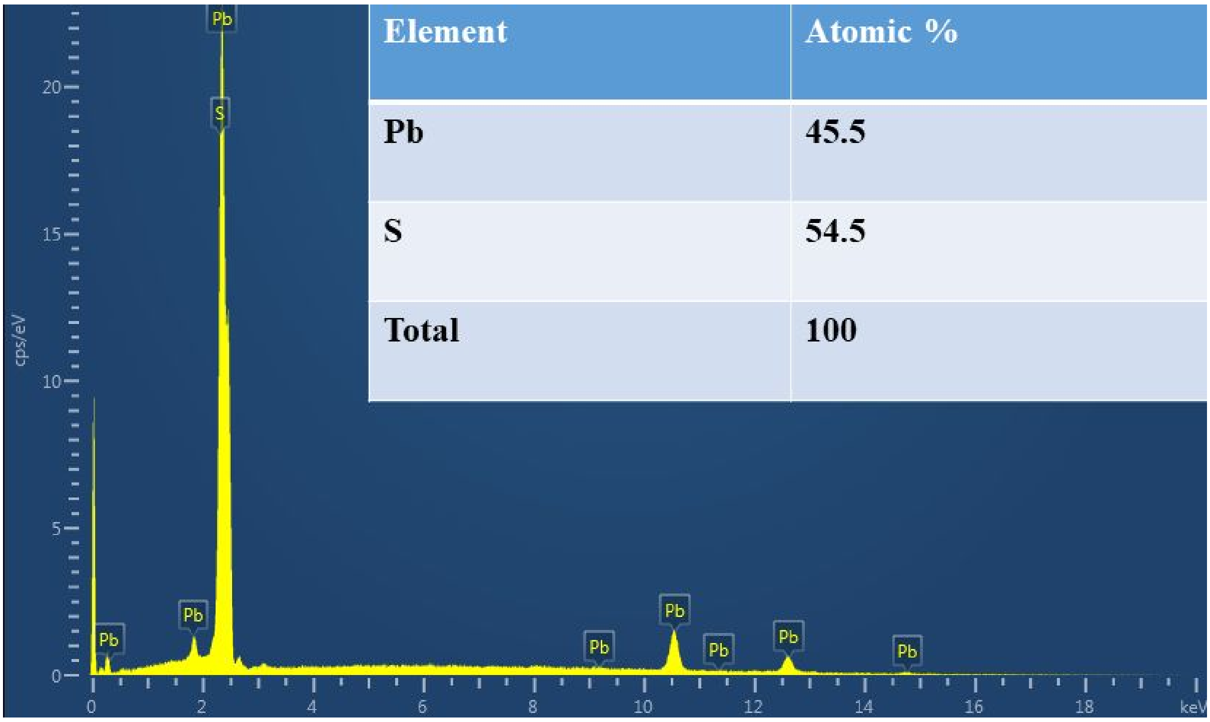

Fig S7 EDX plot of lead sulfide nanocrystals synthesised at 450 °C fo1 h under N_2. Inset is the atomic percentage of Pb and S.

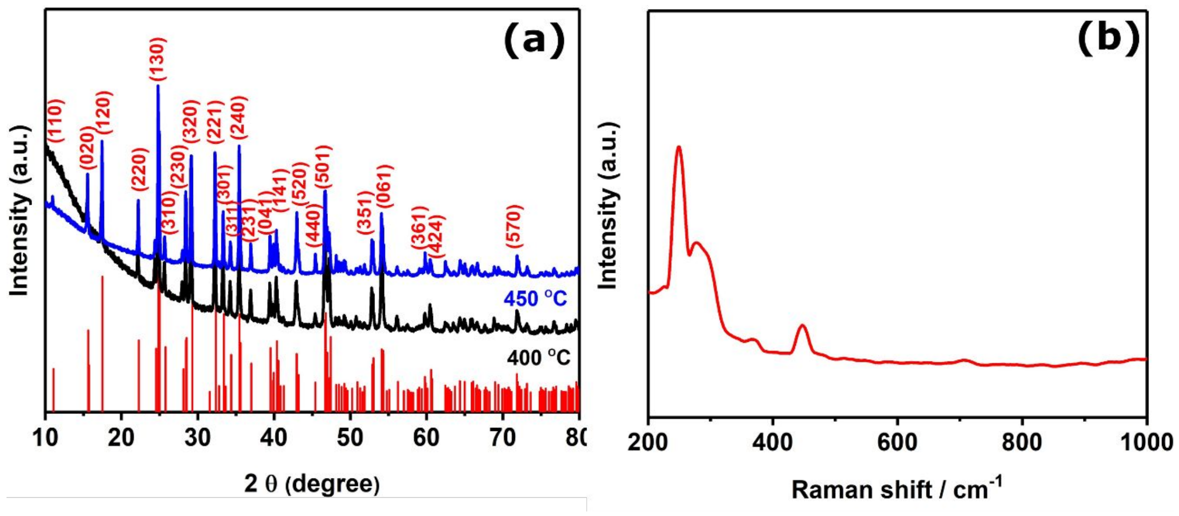

Fig S8: (a) shows p-XRD pattern of the of Sb_2S_3 nanocrystal produced from the decomposition of $Sb(S_2CNEt_2)_3$ complex (**3**) synthesised at 450 °C for 1h under N_2. The XRD pattern correspond to orthorhombic Sb_2S_3 (ICDD No. 01-075-1310) and (b) Raman spectrum of antimony sulfide synthesised at 450 °C for 1 h under N_2.

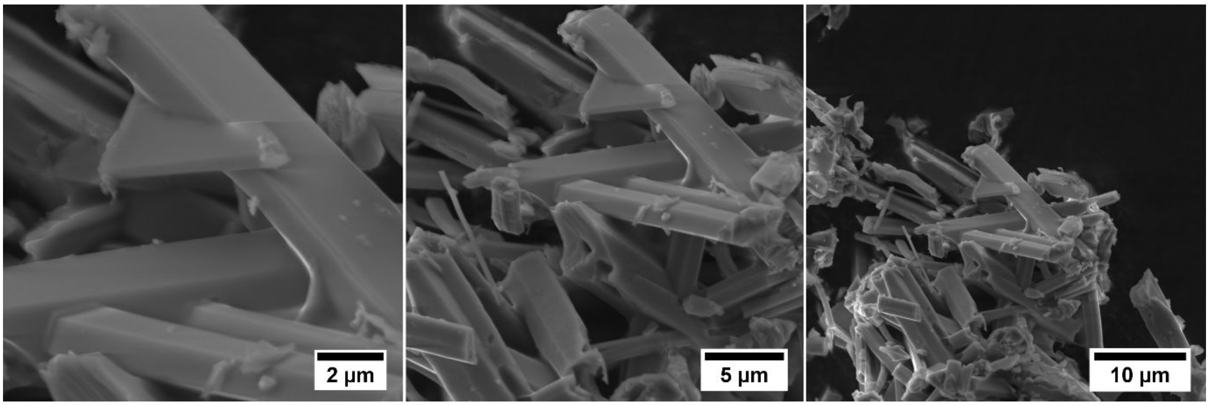

Fig S9 SEM images of as-papered Sb_2S_3 at 450 °C for 1 h under N_2 shows an agglomerated microtubes.

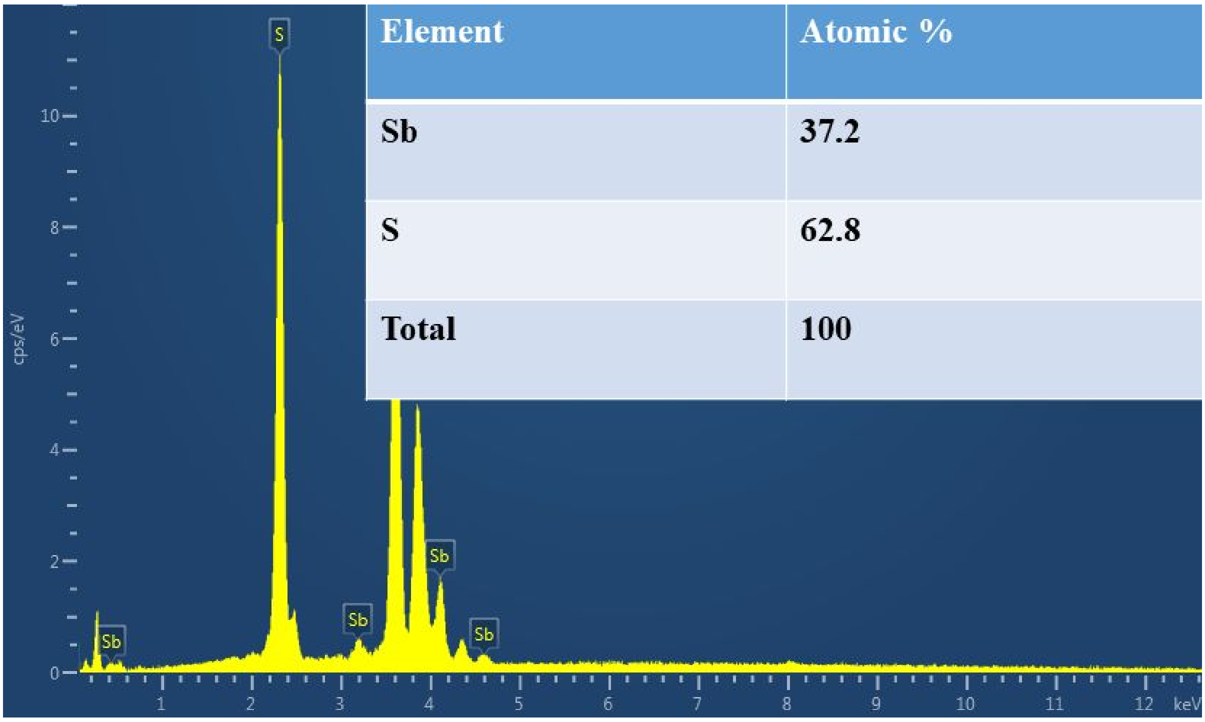

Fig S10 EDX plot of antimony sulfide nanocrystals synthesised at 450 °C for 1 h under N_2. Inset is the atomic percentage of Sb and S.

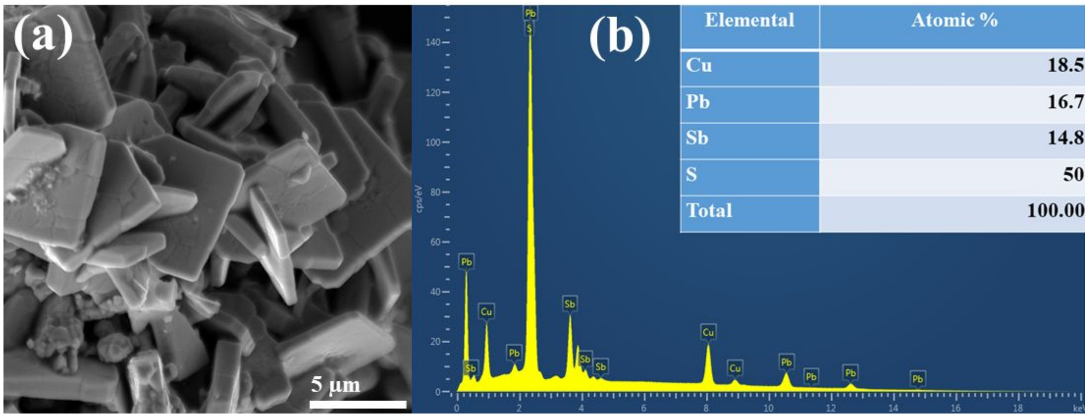

Fig S11 (a) SEM image of bournonite powder synthesised from decomposition of precursors at 500 °C for 1 h. (b) EDX spectrum of bournonite powder. Inset shows the quantification data in at%.

Table S1: The values of 2theta and hkl of p-XRD patterns of bournonite ($CuPbSbS_3$) powder synthesised at 500 °C.

2θ values	hkl value
10.8°	(100)
14.8°	(110)
15.2°	(011)
15.6°	(101)
18.7°	(111)
20.5°	(020)
21.7°	(200)
22.7°	(002)
23.1°	(120)
24.1°	(210)
27.2°	(112)
29.9°	(220)
30.7°	(022)
31.6°	(202)
32.8°	(031)
33.3°	(212)
34.8°	(301)
36.1°	(103)
36.4°	(311)
37.9°	(222)
39.2°	(320)
40.3°	(302)
41.8°	(123)
42.9°	(140)
45.6°	(322)
46.8°	(033)
49.5°	(313)
51.8°	(204)
52.5°	(124)
53.7°	(150)
55.1°	(151)
56.2°	(224)
58.1°	(304)
59.1°	(152)

Table S2: The values of 2theta and hkl of p-XRD patterns of bournonite ($CuPbSbS_3$) Thin film synthesised at 500 °C.

2θ values	hkl value
14.9°	(110)
15.2°	(011)
15.6°	(101)
20.4°	(020)
21.8°	(200)
22.8°	(002)
23.2°	(120)
24.1°	(210)
26.3°	(211)
27.3°	(112)
29.9°	(220)
30.9°	(022)
31.7°	(202)
32.8°	(031)
33.3°	(212)
34.5°	(310)
35.9°	(013)
36.1°	(103)
36.4°	(311)
37.9°	(222)
39.1°	(320)
40.2°	(132)
41.7°	(312)
42.9°	(140)
43.1°	(400)
45.7°	(322)
46.5°	(004)
49.5°	(313)
51.2°	(024)
51.5°	(412)
52.5°	(124)
53.7°	(150)
56.2°	(224)
59.1°	(152)

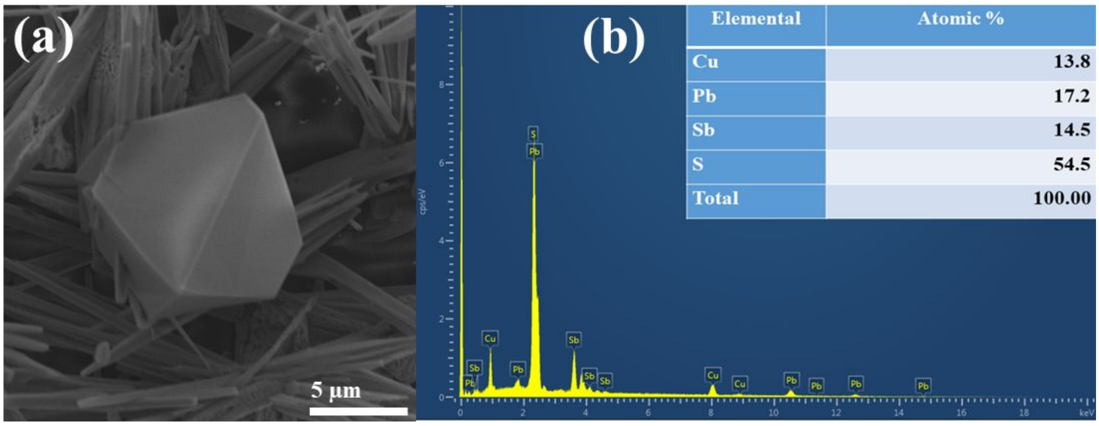

Fig S12 (a) SEM images of bournonite film deposited by Spray-coat-pyrolysis method and heated at 500 °C for 1 h. (b) The EDX plots of bournonite film produced at 500 °C for 1 h. Inset is the compositional data of a bournonite thin film showing the atomic percent of the elements present in the film.

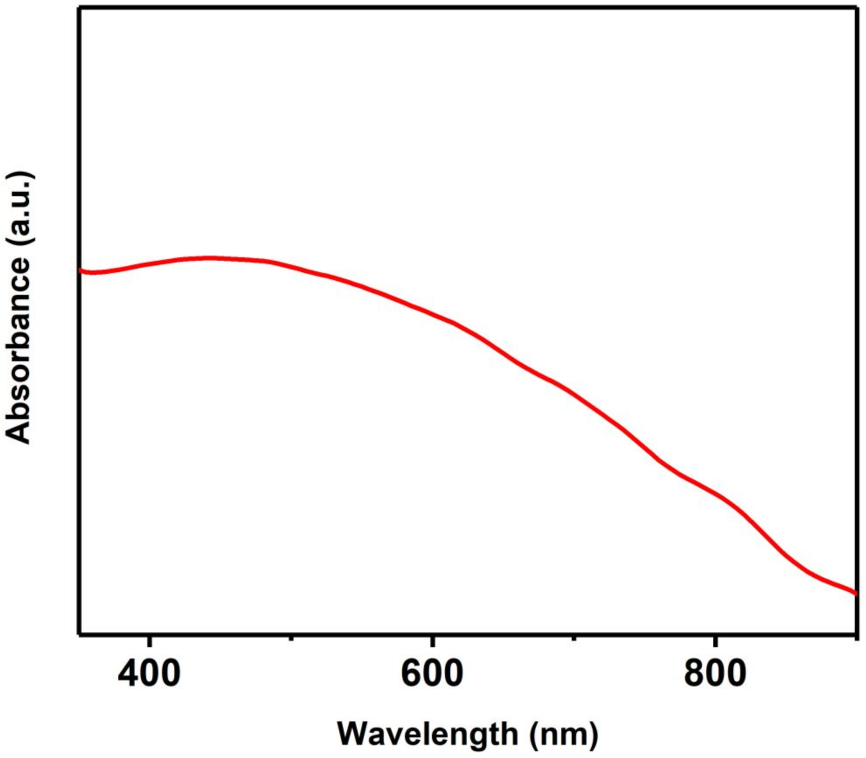

Fig S13: UV-Vis-NIR absorbance spectrum of bournonite film produced at 500 °C for 1 h under N_2.

Conductivity measuremnts:

The resistance (R) of the spray-coated films were measured with a four-point probe system connected to a Keithley 6214B source meter at room temperature. The sheet resistance (R_s) of the films were calculated using obtained resistance values using the equation: $R_s = R \times \pi/ln2$. The thickness (t) of the films were measured by using a Bruker DektakXT surface profiler. Using both R_s and t values, the electrical conductivity of the films were calculated using the equation: conductivity (σ) = 1 / ($R_s \times t$).

Table S3 Electrical properties of bournonite ($CuPbSbS_3$) film deposited on glass substrate via spray-coat-pyrolysis method and heated at 500 °C for 1 h under Argon atmosphere.

Sample	Sheet resistance (GΩ/□)	Film thickness (µm)	Electrical Conductivity (S/m)
$CuPbSbS_3$	2.98	1.44	2.33×10^{-4}
